# New Translational Trends in Personalized Medicine: Autologous Peripheral Blood Stem Cells and Plasma for COVID-19 Patient

**DOI:** 10.3390/jpm12010085

**Published:** 2022-01-10

**Authors:** Mario Giosuè Balzanelli, Pietro Distratis, Rita Lazzaro, Ernesto D’Ettorre, Andrea Nico, Francesco Inchingolo, Gianna Dipalma, Diego Tomassone, Emilio Maria Serlenga, Giancarlo Dalagni, Andrea Ballini, Kieu Cao Diem Nguyen, Ciro Gargiulo Isacco

**Affiliations:** 1 SET-118, Department of Pre-Hospital and Emergency, SG Giuseppe Moscati Hospital, 74010 Taranto, Italy; mario.balzanelli@gmail.com (M.G.B.); distratispietro@gmail.com (P.D.); rita-lazzaro@libero.it (R.L.); 2 Department of Pneumology, SG Giuseppe Moscati Hospital, 74010 Taranto, Italy; ernesto.dettorre@asl.taranto.it (E.D.); andrea.nico@asl.taranto.it (A.N.); giancarlo.dalagni@asl.taranto.it (G.D.); 3 Department of Interdisciplinary Medicine, School of Medicine, University of Bari “Aldo Moro”, 70124 Bari, Italy; francesco.inchingolo@uniba.it (F.I.); giannadipalma@tiscali.it (G.D.); 4 Foundation of Physics Research Center, Celico, 87100 Cosenza, Italy; dietomoh@gmail.com; 5 Department of Blood Banking, SS. Annunziata, 74100 Taranto, Italy; emiliomaria.serlenga@asl.taranto.it; 6 School of Medicine, University of Bari “Aldo Moro”, 70124 Bari, Italy; 7 Department of Precision Medicine, University of Campania “Luigi Vanvitelli”, 80138 Naples, Italy; 8 American Stem Cells Hospital, Ho Chi Minh 70000, Vietnam; drkieukaren@gmail.com

**Keywords:** COVID-19, SARS-CoV-2, arterial blood gas (ABG), laboratory medicine, clinical biochemistry and molecular clinical biology, autologous plasma and peripheral blood stem cells

## Abstract

The COVID-19 pandemic, caused by Severe Acute Respiratory Syndrome Coronavirus 2 (SARS-CoV-2), still remains a severe threat. At the time of writing this paper, the second infectious wave has caused more than 280,000 deaths all over the world. Italy was one of the first countries involved, with more than 200,000 people reported as infected and 30,000 deaths. There are no specific treatments for COVID-19 and the vaccine still remains somehow inconclusive. The world health community is trying to define and share therapeutic protocols in early and advanced clinical stages. However, numbers remain critical with a serious disease rate of 14%, ending with sepsis, acute respiratory distress syndrome (ARDS), multiple organ failure (MOF) and vascular and thromboembolic findings. The mortality rate was estimated within 2–3%, and more than double that for individuals over 65 years old; almost one patient in three dies in the Intensive Care Unit (ICU). Efforts for effective solutions are underway with multiple lines of investigations, and health authorities have reported success treating infected patients with donated plasma from survivors of the illness, the proposed benefit being protective antibodies formed by the survivors. Plasma transfusion, blood and stem cells, either autologous or allograft transplantation, are not novel therapies, and in this short paper, we propose therapeutic autologous plasma and peripheral blood stem cells as a possible treatment for fulminant COVID-19 infection.

## 1. Introduction

Coronaviruses belong to the *Coronaviridae* family and are enveloped into positive-stranded RNA. The SARS-CoV-2 external structure is characterized by an envelope-anchored spike protein, which promotes the virus entrance into host cells. The genome sequencing of viral RNA has revealed that the virus causing COVID-19 is phylogenetically related to the SARS-related coronaviruses, SARS-CoV-1 [1,2]. The angiotensin-converting enzyme 2 (ACE 2) are the main sites used by the spike protein of the SARS-CoV-2 to invade the host [3]. The current situation consists of nearly 300,000 deaths worldwide, with Italy as one of the most affected countries with numbers well over than 200,000 people reported as infected and almost 30,000 deaths. At this point, numbers remain critical, with a serious disease rate of more than 14% and with the majority of patients ending with long-term hospitalization, >40 days, due to general decline as consequences such as sepsis, acute respiratory distress syndrome (ARDS), multiple organ failure (MOF) and disseminated vascular thromboembolism. Though mortality rate is relatively low, within 2–3%, it mainly touches individuals over 65 years old; almost one patient in three dies in the Intensive Care Unit (ICU) [2].

Different solutions have been proposed and the convalescent plasma therapy was surely one of the most applied for prevention and treatment of many infectious diseases for more than a century [1,2,4]. The general consensus sees plasma as a safe and routinely clinical procedure, with a very low risk of complications.

Metanalysis outcomes that compared donated plasma treated vs. untreated SARS patients in terms of mortality rate were significant [5]. Similarly, in COVID-19, authors showed a striking lower mortality rate for COVID-19 ICU patients treated with convalescent plasma vs. untreated patients. Based on such promising results, the clinical trial applications for SARS-CoV-2 treatment is on course for FDA approval [5,6].

However, the main limitation of this procedure is that donated plasma from recovered SARS-CoV-2 patients, who theoretically should have established humoral immunity against the virus, remains poor at neutralizing antibodies, which is crucial in neutralizing and eradicating the pathogen from blood circulation and affected’ pulmonary tissues [1,2,4].

In addition, overall, all patients involved were also treated with multiple other agents including antiviral medications, antibiotics, anticoagulants and anti-histamines, making it difficult to determine whether the observed results could have been related to therapies other than plasma. Still, the percentage of death in patients remains very low compared to the totality of affected patients and, in a few cases, the use of a very distinct agent was reported, such as monoclonal antibody Tocilizumab, an anti-IL-6 that seemed to improve significatively the overall survival rate in severe/critical patients [7].

Comorbidities, age, gender and social status remain a significant point of consideration. Patients affected by preexisting pathologies were calculated to be not less than 70% of the totality at time of entry in hospital. These pathologies included hypertension, overweight, diabetes-2, hypercholesterolemia and thyroiditis. The age, gender and often the low homogeneity of analyzed groups with differences in social and health assistance might also affect therapy outcome [8,9,10]. Therefore, hospitalization time, recovery and degree of resolution of the damaged tissues remain the most solid points for a solid and conclusive comparison.

### 1.1. Physiology of Lung Interstices and Alveolar, the Role of Local Regenerative Mechanisms

The alveolar epithelium is predominantly organized on two types of cell, the alveolar epithelial cell type 1 (AEC1) that compose almost the entire totality of the whole alveolar surface area. This structure shows a thin architecturally complex epithelium, and the cell are specialized for gas exchange. Molecularly the distinctive traits of these cells are defined by the expression of podoplanin and aquaporin-5 markers that play a key role in lung water homeostasis and normal development and functioning of lungs, the lymphatic system and heart. Without either aquaporin-5 or podoplanin, differentiation of type-I pneumocytes is inhibited, leading to the accumulation of underdeveloped lung alveoli [11,12,13]. It was also postulated that both proteins may have a role in regulating the proliferative potential of lung cells, which in turn trigger the differentiation into type-I pneumocytes. Intriguingly, a small fraction of AEC1s were seen to be able to express HOPX, a trait that indicates a further sub-group capable of giving rise to AEC2s [11,12,13,14,15].

In addition, the existence of unconventional epithelial stem cell populations in the distal lung was supported by Chapman and colleagues; these integrin α6β4-positive alveolar stem cells are presumed to play a part in reconstituting an injured alveolar epithelium [16]. Mice following severe influenza infection were confirmed to have regenerated and well re-organized tissue thanks to the presence of these distal airway stem cells with reparative abilities. These special cells were seen to be able to express TRP63 and KRT5 basal cell markers, and were able to generate differentiated alveolar and bronchiolar epithelium following flu damages [17].

However, it remains unclear as to how peripheral and systemic stem cells may eventually become involved in distal alveolar repair mechanisms. In many of the studies using stem cells (SCs), and in particular mesenchymal stem cells (MSCs) either from bone marrow (BM) or peripheral blood (PB), we observed derived epithelial cell growth and proliferation after some form of lung tissue injury, consequent of acute respiratory distress syndrome (ARDS) and chronic obstructive pulmonary disease (COPD) [17].

### 1.2. The Rationale Uses of Autologous Peripheral Blood Plasma and Stem Cells in COVID-19

It is still unclear which of the MSCs, BM or PB subpopulations are involved into the differentiation process to lung pneumocytes, since both sources were shown to contain at least two different populations of stem cells, each of which were capable of both self-renewal and keeping their differentiation capacity towards multiple phenotypes. Nevertheless, both BM and PB were shown to contain neural stem cells (NSCs), embryonic-like stem cells (ESCs) and the MSCs [18,19,20]. Therefore, a co-existing mechanism for plasticity could be based on the “fusion concept” which proposes a kind of combination between stem cells and non-hematopoietic cells lineage that may eventually induce the production of a heterokaryon. Technically, the heterokaryon, which normally refers to multinucleate cell that contains genetically different nuclei, in this case is indicative of a sort of reprogramming mechanism, a repair process of multi-lineage potential to cells with a previously restricted cellular fate. This is a conclusion also based on stem cells reverse transcriptase enzyme that allows the differentiating shift to mature cell type [18,19,20,21,22,23,24].

Through this mechanism, the stem cell can absorb the mature cell micro-vesicles containing mRNA that is successively released into the cytoplasm that can be detected either in the epithelial cell-specific mRNA or by the protein translated from this mRNA. A further possibility is the presence of circulating epithelial progenitor cells in the BM and PB capable of engraftment as epithelial cells generating new engrafted tissues. It is also possible that the engraftment of BM-derived cells occurs via multiple different mechanisms [25,26,27].

In addition, the choice for the uses of autologous plasma in this emergency follows the results obtained by our team few years ago and successively published during the years 2015–2016. The findings revealed that PB plasma either obtained by centrifugation or by natural sedimentation showed the presence of different sub-groups of pluripotent and multipotent stem cells [28,29]. The withdrawn plasma obtained was carefully layered with Ficoll–Paque and centrifuged. The mononucleated cell layer concentration was quantified in a number between a few hundred thousand and a few million. To assess cell identity and phenotype, cells were cultured up to 12 days and analyzed by RT-PCR and flow-cytometry. The RT-PCR and flow-cytometry results both confirmed the expression of multipotent, pluripotent and totipotent markers of adherent and non-adherent mononucleated cells such as Oct4, Sox2, OCN, Nestin, Nanog, DMP and CD44, CD73, CD90, CD133, CD 34, CD45, CD14, Nestin, SSEA3 and Tra1. In addition, the team was able to confirm (using two group control and study) the presence of 14 hormones (among them testosterone, E2, Progesterone, cortisol, etc.) and the expression of cytokines and interleukins (TNFα, IL-6, IFNy and IL-2) within the extracellular matrix components of the stem cell medium culture [4,19,20,29,30].

Another important feature of the PB stem cells is their ability to secrete important growth factors such as the platelet-derived growth factor (PDGF), the vascular endothelial growth factor (VEGF), the fibroblast growth factor (FGF), and the transforming growth factor (TGFβ) decisive in all regeneration process contributing to angiogenesis, self-repairing mechanism and stem cell viability on injured tissues [29,30,31,32,33].

In order to preliminarily evaluate the importance of autologous plasma and stem cell transfusions in COVID-19 critical patients, we presented the resolution and post-hospitalization recovery time of this unique case report from 118 Pre-Hospital and Emergency Department and Pneumology Department of “SG Moscati Hospital” of Taranto City in Italy. The totality of the hospitalized patients between the period of September 2020 and September 2021 was well over 1500 patients. All patients, including our case report’s patient, received a common therapeutic protocol as described in Table 2.

## 2. Materials and Methods

Here, we present a case report of a 56-year-old man who tested positive to SARS-CoV-2 on 17 November 2020. He was admitted to “SG Moscati” Hospital of Taranto Italy on 18 November 2020 in the Pneumology Department due to an alarming worsening condition that included also a severe event of kidney anuresis. Written and verbal information was given to the patient before enrollment, and written informed consent was obtained. The study was conducted in full accordance with the World Medical Association Declaration of Helsinki on experimentation involving human subjects, as revised in 2008. This study has received the approval of: The Independent Medical Ethics Committee of Brindisi, Protocol N. 44941-R.C.E. 81/20. The patient, admitted to Intensive ICU on November the 18th was overweight and presenting clinical manifestations included hypertension, pre-diabetic with a chronic clinical history of asthma (Table 1).

**Figure 1:**
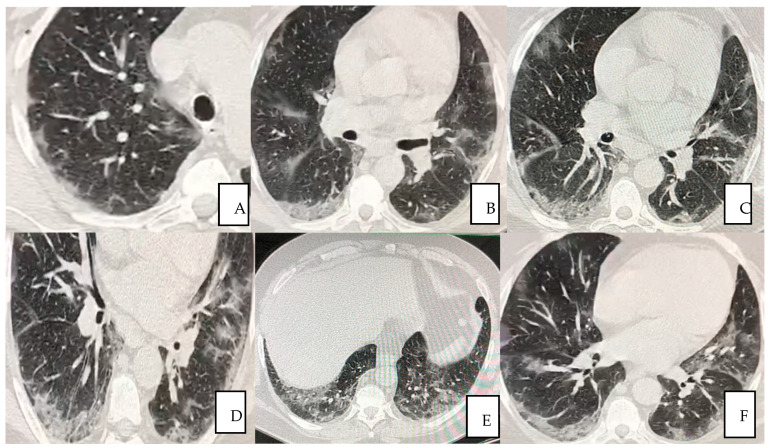
(**A**–**F**) A 56-year-old man presented with fever for 3 days, accompanied by anuria, asthenia, general malaise and positive to COVID-19 (**A**–**E**) Pulmonary HRCT obtained on 18 November 2020 showed multiple peripheral patchy ground glass opacities bilaterally being the lower lobes the most involved areas in (**F**).

The first pulmonary high-resolution computed tomography (HRCT) scan at the admission on November the 18th revealed extensive bilateral damages as shown in Figure 1A–F. The CT scan results at the time of the admission often confirmed severe deteriorated lung structures and functionality.

The patient tested negative to naso-pharyngeal swab (real-time PCR) ten days after the admission, on 29 November 2020. The treatment by PB plasma/SCs was commenced at day one at time of the admission.

### The In Vivo Plasma Procedure

In the in vivo procedure, PB was collected in 10 vacutainer tubes with lithium Eparine (BD Vacutainer^®^, Plymouth, UK) and left to rest until the complete separation of plasma and red part. Plasma was then gently removed from each vacuum tube manually by using a syringe. Once all plasma was collected, it was added to 0.2 mL of vitamin C (injectable solution, Bayer, Italy), 0.2 mL N acetyl-cysteine (NAC) (Flumicil injectable solution, Zambon, Italy), 0.5 mL vitamin D (D-base, injectable solution, Abiogen-Pharma, Italy) and 0.2 mL adenosine (Krenosin, injectable solution, Sanofi, Italy). The whole compound was then left to rest for about ten minutes and injected under the skin in multiple sites on the abdomen (around the navel), on the chest and along the sternum area. The entire procedure was performed for a total of 12 times, executed once per week.

## 3. Results

**Figure 2:**
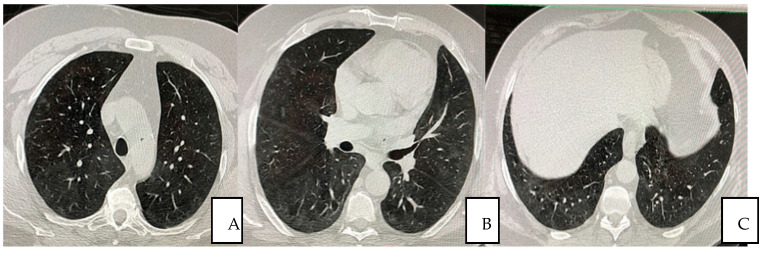
(**A**–**C**) Pulmonary HRCT scan obtained on day 35 from symptom onset (23 December 2020) shows almost complete resolution of the initial presentation.

The patient was treated with autologous plasma and stem cells. He was not a smoker; he was overweight, with pre-existing medical conditions such as asthma, seasonal allergy and hypertension. He received the same protocol treatment as the other hospitalized patients as per the “SG Moscati Hospital” guidelines, composed of antiviral agent, steroid, anticoagulant and antibiotic agents. The protocol previewed 12 applications until the complete resolution of the patient. Autologous plasma administered between the day 1 and 22 days after the admission showed no collaterals, rejection or adverse events. The viral load was undetectable at day 10 in the hospital and any severe side effects were observed thoroughly the whole treatment period afterwards. At day 12, the patient was dismissed and the treatment continued on weekly bases each time measured by Complete Blood Count (CBC) analysis. Typical CT imaging of COVID-19 includes bilateral, apical, parenchymal, peripheral, and basal predominant ground-glass opacity and consolidation. The CT shows that at day 35, with a total of five plasma transfusions, the damages had been reduced by 98% (Figure 2A–C).

**Figure 3:**
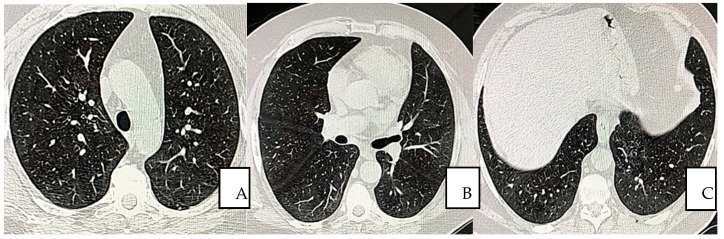
(**A**–**C**) Pulmonary non-contrast enhanced chest CT scan obtained on day 60 from symptom onset (16 February 2021), shows continued and completed resolution without residuals. Parenchymal, mediastinum, and peripheral (**A**,**B**), lower lobe I opacities and bands are not observed (**A**,**B**).

The overall condition improved within 10 days after the first transfusion. The HRCT values became completely negative at day 60 after the 12th transfusion (Figure 3A–C).

**Figure 4:**
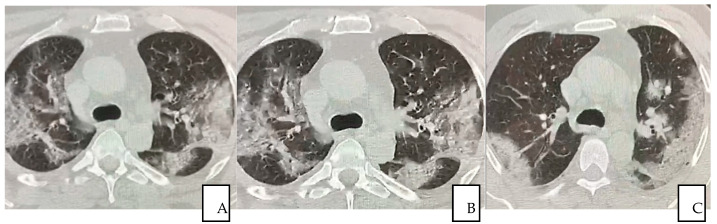
(**A**–**C**) A 56-year-old man from “SG Moscati Hospital”, Taranto, presented with fever, cough, and chest pain. Pulmonary HRCT at presentation on 3 March 2020 showed extensive peripheral predominant ground-glass opacities in both upper lobes (**A**); On 26 March 2020, a follow-up HRCT showed similar anomalous ground glass opacities (**B**) HRCT obtained on 14 May 2020 showed organizing changes with decrease in extent of the ground-glass opacities and increasing parenchymal consolidations (**C**).

Serial chest CT scan during recovery from COVID-19 were performed to evaluate lung abnormalities displayed as ground-glass opacity with the development of white net pattern and increased consolidation (i.e., more extensive lung involvement), and following resolution. The severity of lung damage and the differences between patients who recovered with conventional treatment and the patient received additional autologous plasma and stem cells treatment were assessed and described by comparing CT scan images. It is worth emphasizing that patients showed interstitial lung damages even long after COVID-19 resolved (12 months), as reported in this study (Figure 4A–C).

The arterial blood gas (ABG) parameters at the admission revealed a combination of hypoxic and hypocapnic state with an evident alkalosis, hypocalcemia and hypokalemia. The pH was 7.499 accompanied by PaO_2_ 65.0 mmHg, PaCO_2_ 33.0 mmHg, FO_2_Hb 93.4%, Ca^++^ 1.10 mmol/L and K^+^ 3.43 mmol/L prior to transfusion, levels that improved within 12 days after transfusion and increased substantially on the 12th plasma treatment. Body temperature ranged from 37.6 °C to 39.0 °C before plasma transfusion and declined to the normal range on the third day after the first transfusion (Table 2 and Table 3).

## 4. Discussion

Alongside an antiviral treatment and, virus-specific neutralizing antibody, which could accelerate virus clearance and prevent entry into target cells, the autologous plasma/SCs similarly to the donor’s plasma may serve as the main mechanism to contrast the virus aggression and infection progression [34]. In this case report, a critically ill 56-year-old patient who tested positive for COVID-19 received autologous plasma and SCs obtained by peripheral blood. The plasma/SCs were transfused on the same day, which helps preserving the structural composition and activity of the plasma. As assessed by naso-pharyngeal swab (real-time PCR) the viral load declined within 10 days, symptoms and clinical condition started improving since the first dose of plasma, as intermittently confirmed by arterial blood gas analysis (ABG), blood test and chest imaging. At day 3, mechanical ventilation (CPAP) and extracorporeal membrane oxygenation (ECMO) were no longer required for continuing with nasal cannula for oxygen support. The average time for >90% of lung resolution in post-COVID-19 dismissed patients is calculated to be approximately from 5 to 6 months to 1 year. In addition, once compared to patients who underwent conventional treatment, the patients went back to his daily activities as soon as he was discharged by the hospital. In addition to viral neutralizing immunity, acceleration of infected cell clearance by plasma/SCs has also been found in an in vivo study of HIV-1 virus [4,35]. In the current study, the patient received the entire anti-COVID-19 treatment protocol during his hospitalized period as described in Table 2. The patient was the only one authorized to receive the current treatment among a large number of in-bed patients (>1000) hospitalized in the Pneumology Department. Finally, yet importantly, the home-care prescription based on the use of vitamin C, vitamin D and NAC as cell and plasma activators may also open up the opportunity of using an additional support in the stem cell therapy in vivo against these types of aggressive infections. The importance of vitamin C prescription is based on preceding reports regarding its advantages on supporting bone marrow stem cells proliferation, growth and activation. Similarly, vitamin D3 as pro-hormone has been confirmed in reducing earlier apoptosis of stem cells, stabilizing their mitochondrial activity and improving the inner metabolism. The rationale use of N-Acetyl-Cysteine (NAC) is based on its anti-oxidant properties via the glutathione synthesis mechanism on either cells or tissues. Previous reports confirmed the NAC antioxidant capacity in decreasing ROS levels in the BM of NOD/SCID mice. The NAC-treated models displayed a 10.8-fold increase in hematopoietic engraftment in the injected tibiae. Studies on humans also confirmed the trend NAC displayed a significant increase in human hematopoietic stem cells (HSC) engraftment and hematopoietic differentiation [36,37,38,39]. New understandings of vitamin C, vitamin D and NAC in a combined plan treatment and their advances in metabolomics, transcriptomics, epigenetics, in relation to their ability to control stem cells oxidative stress and possible telomere enhancement might be promising tools to achieve better clinical outcomes in clinical procedures.

To resume, over 1000 patients were hospitalized during the period from November 2020 to January 2021. The patient was the only one to receive the plasma/SC treatment; he was the only case discharged within 10 days, and he was the only one to recover from the lung damage within 2 months. Resistance to viral infections is well known as one of the typical phenotypic features of stem cells and reflects their unique bio/physiological phenotype. The explanation relies on their capability of using antiviral RNA interference (RNAi), a kind of the interferon-independent repression of endogenous retroviruses and intrinsic expression of antiviral stimulated genes (OCT4, NANOG, SOX2, Tra1), that turn on whenever a virus attempts to enter inside a stem cell. Recent evidence suggests that mammalian germ cells and thus the ESCs may retain the ability to process long double stranded RNA (dsRNAs) [40]. The small RNA pool in embrionic stem cells (ESCs), contains small interfering RNAs (siRNAs), which is a very peculiar trait of stem cells, as somatic cells completely lack of siRNA, derived from the double strand RNAa (dsRNAs) produced by endogenous retrotransposons. Each single siRNA tends to form a complex structure that functions as a virus RNA suppressor mechanism, the RNA-induced silencing complex (RISC). Therefore, due to the stem cells’ capacity for producing siRNAs, pluripotent stem cells may rely on RNAi to fight viral infection [40,41]. The evidence of a non-classical antivirus ability of stem cells are confirmed indirectly from works performed on induced-pluripotent stem cells (iPSCs). It is well known that iPSCs are obtained by mature firbroblasts by inserting into them pluripotency genes OCT4, NANOG, SOX2, Tra1. These mature fibroblasts as all somatic cells use the interferone1 (IFN1) pathway to respond to virus attack. However, as soon as fibroblasts cells are converted to iPSCs, they immediately fail to engage the IFN1 mechanism [42].

Though SCs in general are a striking topic, clinical efficacy is often variable and unclear depending on both donor and receiver internal condition. Distinctive traits and patterns are mainly linked to their source, for example, from embryo, bone marrow, peripheral blood, placenta, umbilical cord, fat tissue, etc. Differences also depend on the phenotype such as totipotent, pluripotent and multipotent. In addition, the internal microenvironment may influence the outcome and success of the therapy; subjects with metabolic disorders may give some limitation to stem cells growth and development and the quality of their plasma would be often low. The age and preexisting condition are surely crucial factors that determine the fate of the SCs once injected, cells from younger donors showed increased expression of a variety of growth factors and cell mediators (MCAM, VCAM-1, ALCAM, PDGFRβ, PDL-1, Thy1 and CD71) together with a lower expression of IL-6 when co-cultured with activated T cells. In general, there are few distinctions between male and female. SCs from females expressed more IFN-γR1 and IL-6β, higher estradiol (E2), higher progesterone and showed higher suppressive ability towards T cell proliferation, which may explain males’ higher susceptibility to inflammation and infection [30,34].

## 5. Conclusions

To the best of our knowledge, no equal translational studies on SARS-CoV-2 patients are available. Those study’s outcomes indicated the success in decreasing the virus aggression limiting the progressive tissue injuries and damages as well as important clinical improvements, such as the comprehensive restoration of breathing capacity without the use of external support. After completing the intradermal injection, the CT-scan confirmed the recovery of lung bilateral infiltrates. However, we are well aware of the presence of the current study’s limitations. First, this was a case report. Second, we are aware that we need to better clarify the plasma/SCs role in both regenerative and immune modulation process. Third, the patient was treated with multiple agent therapy (including antiviral medication) which makes it difficult to assess with a degree of certainty what and which worked the most. Fourth, it is difficult to assess whether this approach would reduce case fatality rates. However, the optimal dose and time point, as well as the clinical benefit of the therapy, needs further investigation in larger, well-controlled trials.

## Figures and Tables

**Table 1:** Complete Blood Count (CBC) and arterial blood gas (ABG) parameters at the time of the admission on 18 November 2020.

**The CBC Parameters at the Time of the Admission on 18 November 2020**	***Clinical Laboratory Values***
White cell count (WCC)	11.66 (normal range 3.5–10)
Neutrophils	89.6% (normal range 35–75)
Lymphocytes	7.5% (normal range 20–55)
C-reactive Protein (CRP)	80.4 mg/L (normal range up to 3.5)
Erythrocyte sedimentation rate (ESR)	88 mm/h (normal range 1–10)
Fibrinogen	643 mg/dL (normal range 200–400)
IL-6	66.9 pg/mL (normal range up to 3)
Vitamin D	14.1 ng/mL (<30 insuff; 30–50 suff; >50–100 optimal)
**The ABG Parameters (Arterial Blood Gas) 18 November 2020**	***Clinical Laboratory Values***
pH	7.5 (normal range 7.35–7.45)
PaCO_2_	33 mmHg (normal range 35–45)
PaO_2_	65% mmHg (normal range 75–100)
FO_2_Hb	93.4% (normal range 94–97)
Glucose	119 mg/dL (normal range 80–115)
Lac	1.24 mmol/L (normal range 0.50–2.00)
K^+^	3.43 mmol/L (normal range 3.5–5.3)
C^++^	1.10 mmol/L (normal range 1.12–1.32)
**Pulmonary High-resolution Computed tomography (HRCT) ** **18 November 2020**	***Diagnosis***
	Positive with ground-glass opacities indicating a bilateral, multisegmental, mid-basal and interstitial involvement, suggestive of 35% injury of the total lung surface, as shown in Figure 1.

**Table 2:** Hospital and homecare therapy.

Home Oral Therapy	*Drug Posology*
Ramipril (ACE inhibitor)	10 mg, 1 cpr day
Fleiderina (anti-atrial fibrillation)	200 mg, 1 cpr day
Lobivon (anti-hypertension)	5 mg, 1 cpr day
**Hospital therapy**	***Timeline***
Kcl retard (oral) 600 mg	from 18 to 25 November
Norvasc (oral) 5 mg 1 cp	from 20 November in replacing Lobivon
Clexane (ID) 6000 1 fl day	from 20 November to 1 December
Pantorc (IV) 40 mg 1 fl day	from 18 November to 1 December
Decadron (IV) 8 mg 1 fl × 2	from 18 to 26 November
Decadron (IV) 4 mg 1 fl × 2	from 27 November to 1 December
Rocefin (IV) 2 g 1 fl	from 18 November to 1 December
Veklury (IV) 100 mg 2 fl + S.F. 250 mL in 2 h	from 15 November to 19 November
Veklury (IV) 100 mg 1 fl + S.F. 250 mL in 2 h	from 20 November to 23 November
O_2_ in CPAP (peep 10 cm H_2_O) + FiO_2_	***O_2_ Supplementation (%)***
	60% (day 18 November) 50% (days from 20 to 25 November) 40% (day from 26 to 29 November) On 30 November, O_2_ supplementation ended.

**Table 3:** CBC and ABG parameters at the time of the dismissing 1 December 2020.

**The CBC Parameters on Dismissing 1 December 2020**	***Clinical Laboratory Values ***
White cell count (WCC)	5.14 (normal range 3.5–10)
Neutrophils	82.8% (normal range 35–75)
Lymphocytes	711.02% (normal range 20–55)
C-reactive protein (CRP)	2.9 mg/L (normal range up to 3.5)
Erythrocyte sedimentation rate (ESR)	18 mm/h (normal range 1–10)
Fibrinogen	458 mg/dL (normal range 200–400)
IL-6	2.7 pg/mL (normal range up to 3)
Vitamin D	42.1 ng/mL (<30 insuff; 30–50 suff.; >50–100 optimal)
**The ABG parameters (arterial blood gas) 1 December 2020**	***Clinical Laboratory Values ***
pH	7.4 (normal range 7.35–7.45)
PaCO_2_	35.8 mmHg (normal range 35–45)
PaO_2_	85 mmHg (normal range 75–100)
FO_2_Hb	95.3% mg/dL (normal range 94–97)
Glucose	240 mg/dL (normal range 80–115)
Lac	3.38 mmol/L (normal range 0.50–2.00)
K^+^	3.80 mmol/L (normal range 3.5–5.3)
C^++^	1.23 mmol/L (normal range 1.12–1.32)
**Pulmonary HRCT** **15 December 2020**	***Diagnosis***
	In complete resolution any ground-glass opacities were seen indicating healing process of 98%.

## Data Availability

Data are contained within the article.
